# Maize miRNAs and their putative target genes involved in chilling stress response in 5-day old seedlings

**DOI:** 10.1186/s12864-024-10403-1

**Published:** 2024-05-15

**Authors:** Manja Božić, Dragana Ignjatović Micić, Nenad Delić, Ana Nikolić

**Affiliations:** Laboratory for Molecular Genetics and Physiology, Research and Development Department, Maize Research Institute Zemun Polje, Belgrade, Serbia

**Keywords:** miRNA, Chilling stress, Maize, 5d-old seedlings, Target genes

## Abstract

**Background:**

In the context of early sowing of maize as a promising adaptation strategy that could significantly reduce the negative effects of climate change, an in-depth understanding of mechanisms underlying plant response to low-temperature stress is demanded. Although microRNAs (miRNAs) have been recognized as key regulators of plant stress response, research on their role in chilling tolerance of maize during early seedling stages is scarce. Therefore, it is of great significance to explore chilling-responsive miRNAs, reveal their expression patterns and associated target genes, as well as to examine the possible functions of the conserved and novel miRNAs. In this study, the role of miRNAs was examined in 5d-old maize seedlings of one tolerant and one sensitive inbred line exposed to chilling (10/8 °C) stress for 6 h and 24 h, by applying high throughput sequencing.

**Results:**

A total of 145 annotated known miRNAs belonging to 30 families and 876 potentially novel miRNAs were identified. Differential expression (DE) analysis between control and stress conditions identified 98 common miRNAs for both genotypes at one time point and eight miRNAs at both time points. Target prediction and enrichment analysis showed that the DE zma-miR396, zma-miR156, zma-miR319, and zma-miR159 miRNAs modulate growth and development. Furthermore, it was found that several other DE miRNAs were involved in abiotic stress response: antioxidative mechanisms (zma-miR398), signal transduction (zma-miR156, zma-miR167, zma-miR169) and regulation of water content (zma-miR164, zma-miR394, zma-miR396). The results underline the zma-miRNAs involvement in the modulation of their target genes expression as an important aspect of the plant’s survival strategy and acclimation to chilling stress conditions.

**Conclusions:**

To our understanding, this is the first study on miRNAs in 5-d old seedlings’ response to chilling stress, providing data on the role of known and novel miRNAs post-transcriptional regulation of expressed genes and contributing a possible platform for further network and functional analysis.

**Supplementary Information:**

The online version contains supplementary material available at 10.1186/s12864-024-10403-1.

## Background

The negative consequences of climate change on crop yield, mainly due to increases in average and extreme temperature, changes in rainfall patterns, and elevated CO_2_ concentrations are already noticeable and the future crop yield projections are being recognized as potentially a major societal concern [1, 2]. By using ensembles of latest-generation crop and climate models, Jägermeyr et al. suggested that climate impacts will emerge earlier than previously projected [3]. At the same time, maize was detected as the most vulnerable crop, because as a C4 crop it has a small capacity to benefit from the elevated CO_2_. Also, it is grown across a wide range of low latitudes which are recognized as the most endangered regions due to current proximity to crop-limiting temperature thresholds**.** As maize is the most important global crop in terms of total production, these projections impose great risks to food security.

However, it is expected that cropping system adaptation and/or better-adapted varieties can considerably reduce climate change impacts [4, 5]. In the latter context, early sowing of chilling tolerant early maturity maize hybrids in temperate regions with hot summers coupled with absence/low rainfall could ensure avoiding the negative effects of drought stress during the flowering period and ensure sustainable yield. In maize, low temperature in the range of 5–15 °C is sufficient to compromise growth from the seedling to the mature stage of development. For this reason, shifting the sowing date forward (sowing at sub-optimal temperatures below 15 °C) requires tolerant hybrids because low temperatures can reduce germination, emergence rate, and seedling vitality. Furthermore, low temperatures affect cell survival, cell division, photosynthetic efficiency, and water transport, subsequently leading to a reduction in plant growth and lower productivity [6].

Disruption of normal cell functions caused by chilling stress demands a brisk and comprehensive reprogramming at the molecular level as the result of transcriptional, post-transcriptional, and translational regulation of stress-responsive genes [7]. MicroRNA (miRNA) are a class of small non-coding RNA molecules approximately 18–24 nucleotides in length which regulate gene expression by cleavage or/and translational inhibition of target mRNA [8]. They control complex regulatory networks, are involved in a broad range of biological processes and are shown to be critical regulators of developmental process and response to abiotic/biotic stress [9]. As they regulate various biological processes by targeting multiple genes simultaneously, miRNAs are considered a promising strategy for complex trait improvement, including yield, biomass production, and stress tolerance [10]. The possibility of harnessing the diverse functions of miRNAs to achieve desirable agronomic traits in important crops, particularly abiotic stress response, was also emphasized by Zhang et al., by reviewing their role and the role of their targets in response to low temperature, high temperature, drought, soil salinity, and heavy metals [8].

It has been shown that miRNAs are involved in the regulation of chilling stress in different plant species. Megha et al. summarized the current understanding of miRNA-mediated modulation of the expression of key genes as well as genetic and regulatory pathways, involved in chilling stress responses in plants [11]. The authors accentuated growing evidence in the literature that miRNAs reprogramming of gene expression is a major defense mechanism in plants enabling them to respond to stresses. Among the miRNAs found to be cold responsive in various plant species are miR319, miR394, miR398 and miR156 [11]. In rice, sugarcane, and cassava, miR319 positively regulates cold tolerance by repressing the expression of two TCP genes. miR394 is highly conserved miRNA in both monocots and dicots, and its involvement in cold stress response was found in *Arabidopsis*. miR398 are known regulators of the antioxidative response in plants, controlling the expression of Cu/Zn superoxide dismutase 1 and 2 (*CSD1* and *CSD2*), shown to affect the tolerance of winter wheat to cold stress. miR156, which are key regulators of squamosa promoter-binding (SPB) like proteins (SPL) expression, were identified as chilling stress-responsive in tomato. Further studies may reveal more detailed functions of miRNAs involved in chilling stress response.

However, despite research done on the role of miRNAs in the chilling response in maize during the V3 stage [12], to our knowledge there are no studies on earlier seedling stages. Considering that early sowing of maize hybrids is a promising adaptation strategy that could significantly reduce the negative effects of climate change, the development of genotypes tolerant to low-temperature stress in the early stages of plant growth and development is of major importance. To achieve this goal, an in-depth understanding of mechanisms underlying plant response to stress is required.

As miRNAs are being recognized as a major defense mechanism in plants enabling them to respond to stress [11], the aim of the experiment was examining the role of miRNAs in the chilling response of young maize seedlings, by applying high throughput sequencing. This study compares the expression of miRNA in the 5-day old seedlings of a chilling-tolerant and a chilling-sensitive maize inbred line, under both optimal and chilling conditions, in order to explore chilling-responsive miRNAs and reveal expression patterns of miRNAs and associated target genes, as well as to examine the possible functions of the conserved and novel miRNAs.

## Methods

### Chilling response assessment of maize inbred lines

Six elite maize inbred lines (L1-L6), parental components of commercial ZP hybrids, developed in Maize Research Institute Zemun Polje, were chosen for the assessment of the chilling stress response. Lines L1 to L3 belong to Lancaster, L4 to Iowa dent, L5 to BSSS/Iowa dent, and L6 to BSSS heterotic groups. With respect to the kernel type, L1 to L4 are dents, and L5 and L6 are semi-dents. According to the breeders' experience in the field regarding chilling stress tolerance, L1 and L6 are considered as highly tolerant genotypes, L2 and L3 as genotypes with low tolerance, while L4 and L5 as intermediately tolerant genotypes.

The experiment was done in three replicates of 10 plants *per* inbred line for each analysis. Seeds were germinated for five days in a growth chamber (MLR-352H-PE Climate Chamber, PHC Europe B.V., The Netherlands), after they were sterilized in 10% sodium hypochlorite (commercial bleach). Germination was performed in the dark at 25/20 °C (12/12 h) and relative humidity of 75%. After reaching the desired growth stage, the 5-day old seedlings were subjected to chilling stress for 24 h at 10/8 °C with a 12/12 h light/dark photoperiod and light intensity up to 700 µmol/m^2^/s. After recording the survival rate (SR), calculated as the percentage of plants surviving the stress treatment, a subset of ten seedlings per genotype was immediately sampled and used for seedling fresh weight (FW_5-d_), radicle (L_rad_) and coleoptile (L_col_) length measurements. The rest of the seedlings were sown in pots containing a mixture of soil and sand (3:1), moved back to the growth chamber, and grown under the optimal temperatures of 25/20 °C, with the same photoperiod, relative humidity, and light intensity as described, for another seven days to recover.

Recovered plants were harvested and used for measuring root (RFW) and shoot (SFW) fresh weight, as well as root (RL) and shoot (SL) length. Dry root (RDW) and shoot (SDW) weights were determined after a 24-h drying period at 110° C in a drying oven. Control plants of all inbred lines were grown under the optimal conditions until the same developmental stage as the treated ones and sampled at the identical time points.

### Chilling treatment of LT and LS lines

Based on the results of the chilling stress response assessment and their statistical significance, two maize inbred lines of contrasting tolerance to chilling were selected for further research. The selection underwent two levels of assessment – following the 24-h stress period and after seven days of recovery. The chosen inbred lines were marked as L_T_, shown to be the most tolerant during the assessment, and L_S_, chosen as the most sensitive of the six lines.

The experimental setup for the low-temperature treatment of L_T_ and L_S_ was the same as explained above – after five days of germination under optimal conditions in the dark, the 5-day old seedlings were exposed to the previously described chilling conditions for 24 h. Sampling for further analyses was done 6 h and 24 h after the start of the treatment. Control plants were grown under optimal conditions in the same time period and sampled at identical time points.

### Total RNA isolation

Total RNA was extracted from 30 maize seedlings *per* inbred line (L_T_ and L_S_) after each time point (6 h and 24 h). The sampled tissue was ground in liquid nitrogen using a pestle and mortar and stored at -80° C until further use. Approximately 100 mg of the frozen tissue was used for the total RNA extraction (GeneJet™ RNA Purification kit, Thermo Scientific, MA, USA). Isolated RNA was further treated with DNase I (Ambion® DNA-free™ kit, Invitrogen, MA, USA), and total RNA concentrations and quality were determined by several methods. Preliminary quantitation was performed with the NanoPhotometer® spectrophotometer (IMPLEN, CA, USA), while agarose gel electrophoresis was utilized to check for RNA degradation and potential contamination. Finally, RNA integrity and quantitation were carried out with the 2100 Bioanalyzer and RNA Nano 6000 Assay Kit (Agilent®, CA, USA). All samples with RIN above seven were chosen for the downstream analysis.

### NGS library preparation and sequencing

Eight samples (four *per* genotype) were used for the library preparation and Illumina sequencing, which were carried out at Novogene Bioinformatics Technology Co., Ltd., in Beijing, China. The L_T_ samples encompassed 6 h and 24 h time points, control (C) and treated (T) plants—L_T_-C-6, L_T_-C-24, L_T_-T-6, and L_T_-T-24, respectively. Accordingly, the L_S_ samples included L_S_-C-6, L_S_-C-24, L_S_-T-6, and L_S_-T-24. Small RNA library construction was carried out using NEBNext® Multiplex Small RNA Library Prep Set for Illumina® (NEB®, USA), adding index codes to attribute sequences to each sample and following the manufacturer’s recommendations. Briefly, small RNA libraries were generated from 3 μg of total RNA by adapter ligation (first the 3', followed by the 5' adapter), first strand cDNA synthesis, PCR amplification, and purification through 8% polyacrylamide gel electrophoresis. Library quality was assessed on the Bioanalyzer 2100 system using DNA High Sensitivity Chips (Agilent®, CA, USA). The clustering of the index-coded samples was performed on a cBot Cluster Generation System using TruSeq SR Cluster Kit v3-cBot-HS (Illumina®, CA, USA) according to the manufacturer’s instructions. After the cluster generation, the single-end 50 bp RNA sequencing was performed on the Novaseq 6000 platform (Illumina®, CA, USA).

### sRNAseq bioinformatics data analysis

The quality of the raw reads was checked using FastQC [13], and then processed through fastp [14] to remove reads containing adapter and poly-N sequences and reads with low quality (Phred score > 30, N% < 10%). Sequencing error rates, Phred and Q-scores, as well as the GC content, were calculated. Additionally, the clean reads of high quality were filtered based on their length and only 18–24 nt reads were used for the downstream analyses. Small RNA tags were mapped onto *Zea mays* B73 NAM reference genome version 5.0 (https://plants.ensembl.org/Zea_mays/Info/Index) using Bowtie 0.12.9 [15] without any mismatches to analyze their expression and distribution on the reference.

Known, conserved miRNAs were identified through the alignment of the mapped sRNA tags to the registered miRNAs in the miRBase database (Release 20, http://www.mirbase.org) [16] using the miRdeep2 software [17]. Sequences with perfect matches were regarded as conserved miRNAs. These detected known miRNAs were also compared to those of different organisms in the miRbase. Novel miRNAs were predicted from the miRNA precursors by applying software tools miREvo [18] and miRdeep2. Both are based on a modified miRDeep algorithm that uses unannotated sRNA tags to predict potentially novel miRNAs, by exploring the secondary, miRNA hairpin structure, the Dicer cleavage site, and the minimum free energy of the unannotated sRNAs. The algorithm also has stringent score cut-offs that significantly limit the possibility of false positives. Tags originating from protein-coding genes, repeat sequences, rRNA, tRNA, snRNA, and snoRNA, were removed by mapping to RepeatMasker (http://www.repeatmasker.org) and the Rfam database [19]. Target genes of the conserved and potentially novel miRNAs have been predicted using the psRobot 1.2 tool [20]. No mismatches were allowed at miRNA positions 2–17 to increase the reliability of the prediction.

Gene Ontology (GO) enrichment analysis of the potential target genes was performed using the GOseq 2.12 Wallenius-based non-central hyper-geometric distribution [21], which could adjust for gene length bias. KOBAS software 3.0 [22] was used to test the statistical enrichment of the target gene candidates in KEGG pathways [23]. Additionally, potential miRNA secondary structures, counts, and first position bias were obtained through miRdeep2.

Differential expression analysis between the control and treatment samples was performed using the DEGseq R package 1.2.2 [24], after previously executed normalization using the TPM algorithm [25]. The p-value was adjusted according to the Bayesian interpretation [26], and the adjusted value, or the q-value was further used. The threshold for significant differential expression was set as q-value < 0.01 and log2 fold change ≥ 1 or ≤ -1.

### Validation of known and novel miRNAs using qRT-PCR

The sequencing results were validated using qRT-PCR to analyze the expression of the selected known and novel miRNAs, as well as their target genes. Five miRNAs were chosen based on their quantity across all eight libraries and differential expression levels: novel_904, zma-miR11970-3p, zma-miR159c-5p, zma-miR166a-5p and zma-miR396c. Chosen corresponding targets genes for these miRNAs were heat stress transcription factor A-2e (*hsfA-2e),* mitochondrial ADP/ATP carrier protein 1 (*aac1)*, *Zea mays* Homeobox-leucine zipper protein, ROC7 (*roc7),* chloroplastic acyl carrier protein 2 (*acp2)* and growth-regulating factors 5 and 8 (*grf5/8),* respectively. Additionally, the expression of several known *Zea mays* cold induced genes (*ZmCOI*) was analyzed using qRT-PCR, to further confirm the chilling response assessment of the two chosen maize inbred lines.

Total RNA was extracted and treated with DNase I, as previously described. Two μg of total RNA were used in cDNA synthesis using the Revert Aid First Strand cDNA synthesis kit with RNase inhibitor (Thermo Scientific™). Real-time PCR analysis was carried out using cyclophilin (*cyp*) as the internal reference gene [27] and three biological replicates for each sample. PCR reactions were performed on a StepOnePlus™ Real-Time PCR System (Applied Biosystems™). The reaction mixture (10 μl) contained 1 × HOT FIREPol® EvaGreen® qPCR Mix Plus (ROX) (Solis BioDyne™), 0.2 μM of each primer (forward and reverse) and 1 μl of template cDNA diluted two times. The thermal cycling conditions included the initial denaturation (95° C for 10 min), followed by 40 cycles of denaturation (95° C for 15 s), primer annealing, and extension (appropriate temperature for 60 s). The primers used are listed in the Additional File 1. Primers for miRNA validation were designed using miRprimer [28], while the target gene primers were designed using Primer 3 (v 0.4.0) online software (http://bioinfo.ut.ee/primer3-0.4.0) and checked in NCBI Primer-BLAST tool (https://www.ncbi.nlm.nih.gov/tools/primer-blast). Relative gene expression was calculated according to Livak and Schmittgen [29] using efficiency correction as in Pfaffl [30]. 

### Statistical analysis

**Fig. 1:**
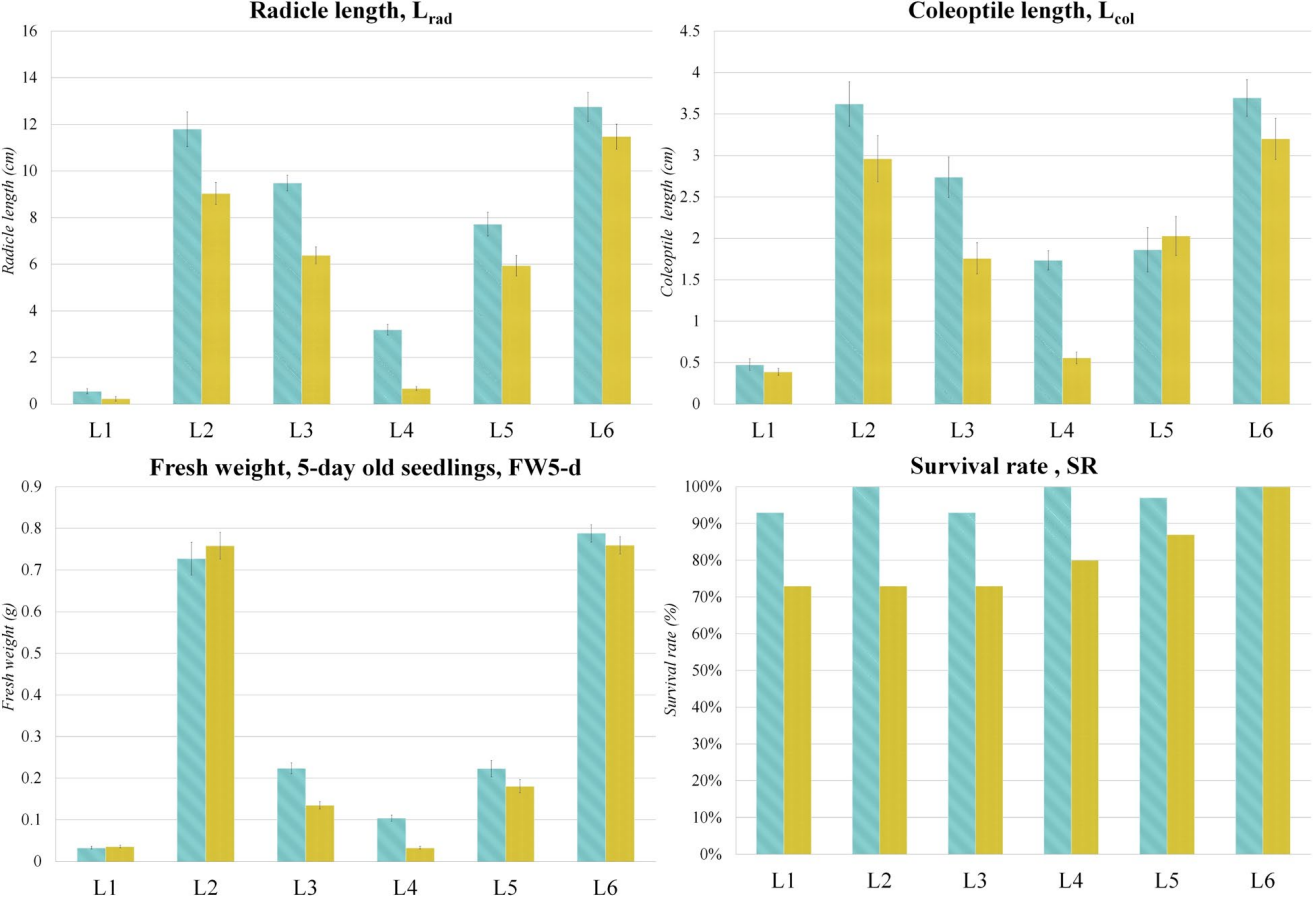
Chilling response assessment of 5-day old seedlings after 24 h stress period. L1 to L6 – six maize inbred lines with different level of chilling tolerance. Control is shown in blue, treatment in yellow. The significance of the difference between the control and treatment of each parameter was determined by t-test and is shown as *** (*p* < 0.001), ** (*p* < 0.01), * (*p* < 0.05), and NS (statistically not significant at *p* < 0.05)

**Fig. 2:**
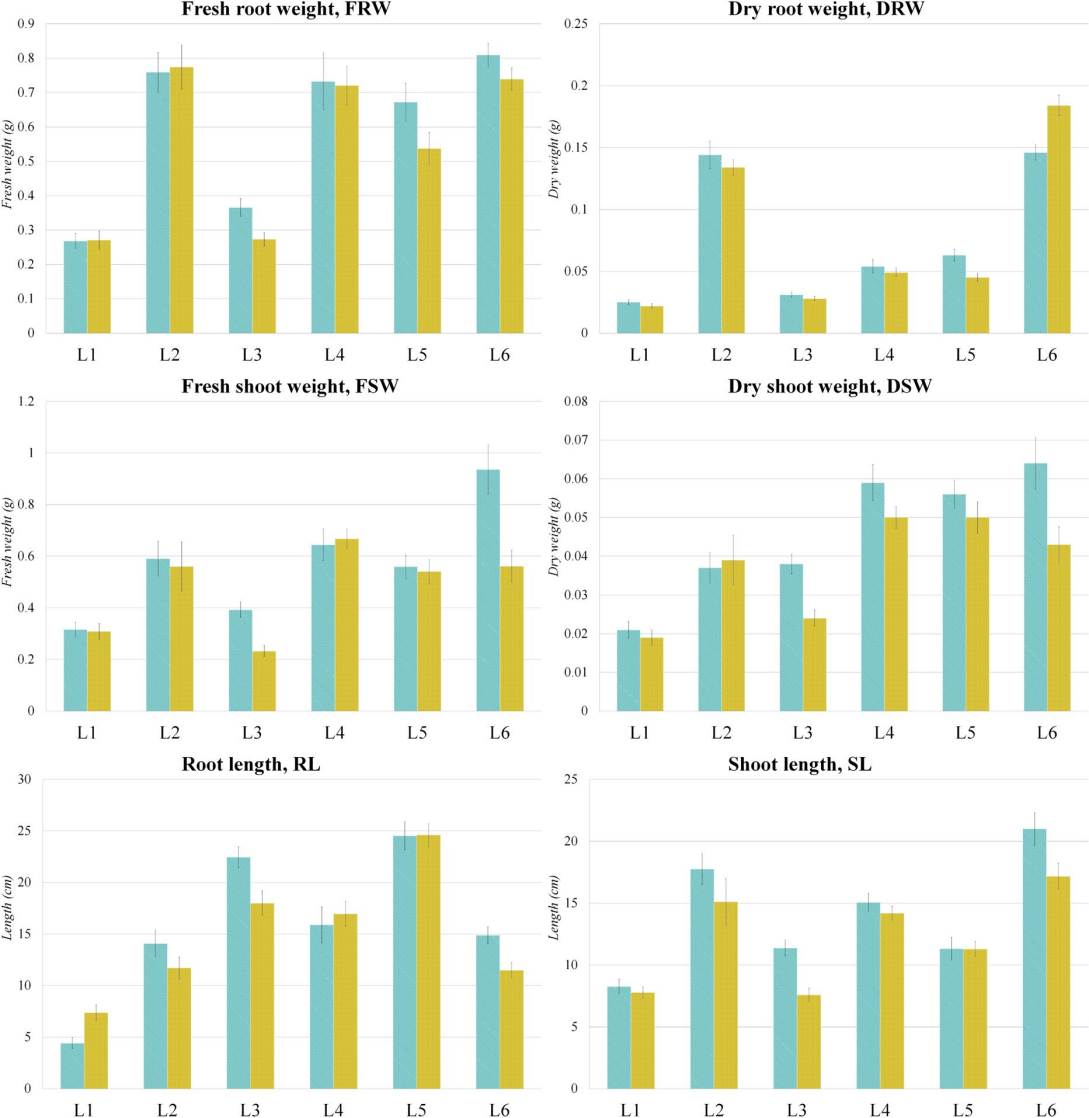
Chilling response assessment after seven days recovery period. L1 to L6 – six maize inbred lines with different levels of chilling tolerance. The control is shown in blue, and the treatment in yellow. The significance of the difference between the control and treatment of each parameter was determined by t-test and is shown as *** (*p* < 0.001), ** (*p* < 0.01), * (*p* < 0.05), and NS (statistically not significant at *p* < 0.05)

All statistical analyses were done using the stats package in R 4.1.2 [31]. The results obtained from the chilling response assessment of maize inbred lines were checked for normality (Shapiro–Wilk and Kolmogorov–Smirnov test) and statistically processed by applying *t-*test with a significance level at *p* < *0.05. t*-test was also carried out for mean comparison with a significance level at *p* < 0.05 for qRT-PCR validation results. Graphs in Figs. 1, 2 and 7 were designed in Microsoft Excel (Windows 10). Cluster analysis, based on the expression patterns of 101 known miRNAs identified in the eight libraries, was performed using normalized read count values and Euclidian distance method, to form both the K-means and hierarchical clusters.

## Results

### Chilling response assessment of maize inbred lines

Chilling stress tolerance of the six inbred lines underwent two levels of assessment – following the 24-h stress period (Fig. 1) and after seven days of recovery (Fig. 2). It can be seen from Fig. 1 that lines L1 and L6 displayed the highest levels of tolerance regarding L_rad_, L_col_ and FW, as all negative effects of the stress were statistically insignificant. However, although insignificant, the percentage of decrease was lower for L6 compared to L1 – 10% vs. 59% for L_rad_, 13.5% vs. 18.4% for L_col_, and 3.7% vs. 9% for FW. Besides, line L6 had higher seed vigor than L1, and it is well known that genotypes with high vigor can cope more efficiently with various stress factors since they evolved complex resistance mechanisms [32]. Moreover, SR of L1 and L6 showed a great difference – L1 showed a 20% decrease, while no change was detected for L6 under the stress. On the other hand, lines L3 and L4 showed the highest level of susceptibility, as the values of all analyzed traits were significantly decreased (*p* < 0.01 or *p* < 0.001). A high decrease of SR (20%) was also detected. The other two lines (L2 and L5) displayed intermediate tolerance with statistically significant changes only for Lrad, but also with 27% and 10% decrease in SR, respectively.

Considering parameters measured after seven days of recovery, L1 displayed better recovery compared to L6 (Fig. 2). The difference between control and treated plants was insignificant for all root and shoot traits measured in L1, except for RL for which a statistically significant increase (*p* < 0.01) was detected. However, a statistically significant increase (*p* < 0.01) in DRW was detected in L6. Still, L6 was chosen as the tolerant inbred line for further studies due to its better performance at the 5-day stage, considering that shifting sowing data backwards requires genotypes tolerant to low temperatures during germination. The L3 line was chosen as the most susceptible line, as it had the poorest performance in both 5-day and recovery stages. From this point onward, L3 will be labeled as L_S_ and L6 as L_T_.

### Identification of known and potentially novel miRNAs

**Fig. 3:**
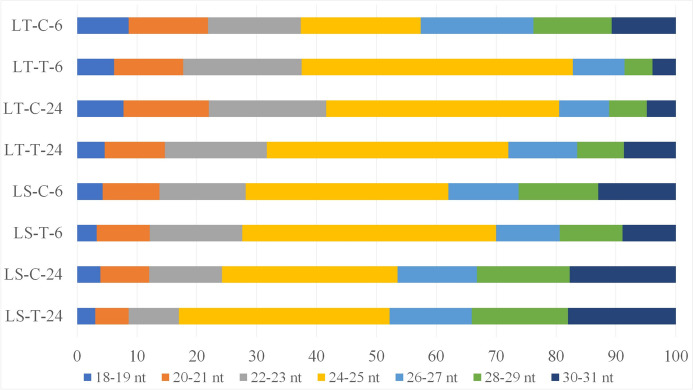
Size distribution of detected sRNAs. sRNAs are sorted into groups of 18–19 nt, 20–21 nt, 22–23 nt, 24–25 nt, 26–27 nt, 28–29 nt and 30–31 nt

On average, 21.3 million clean reads were generated from the sequencing the eight sRNA libraries and 13.9 million were 18–30 nt in length. Size distribution of the reads (Fig. 3) was similar across all eight libraries and the majority of reads were 20–24 nt long (on average 67.9%), with the most abundant being 24 nt long reads (on average 29.97% of all 18–30 nt reads). Most of the clean reads (81.39%) were successfully mapped sRNA reads, and 56.9% were unique sRNA reads. For annotation purposes, the sRNAs were grouped into several classes, shown for the total and unique reads (Additional File 2).

**Fig. 4:**
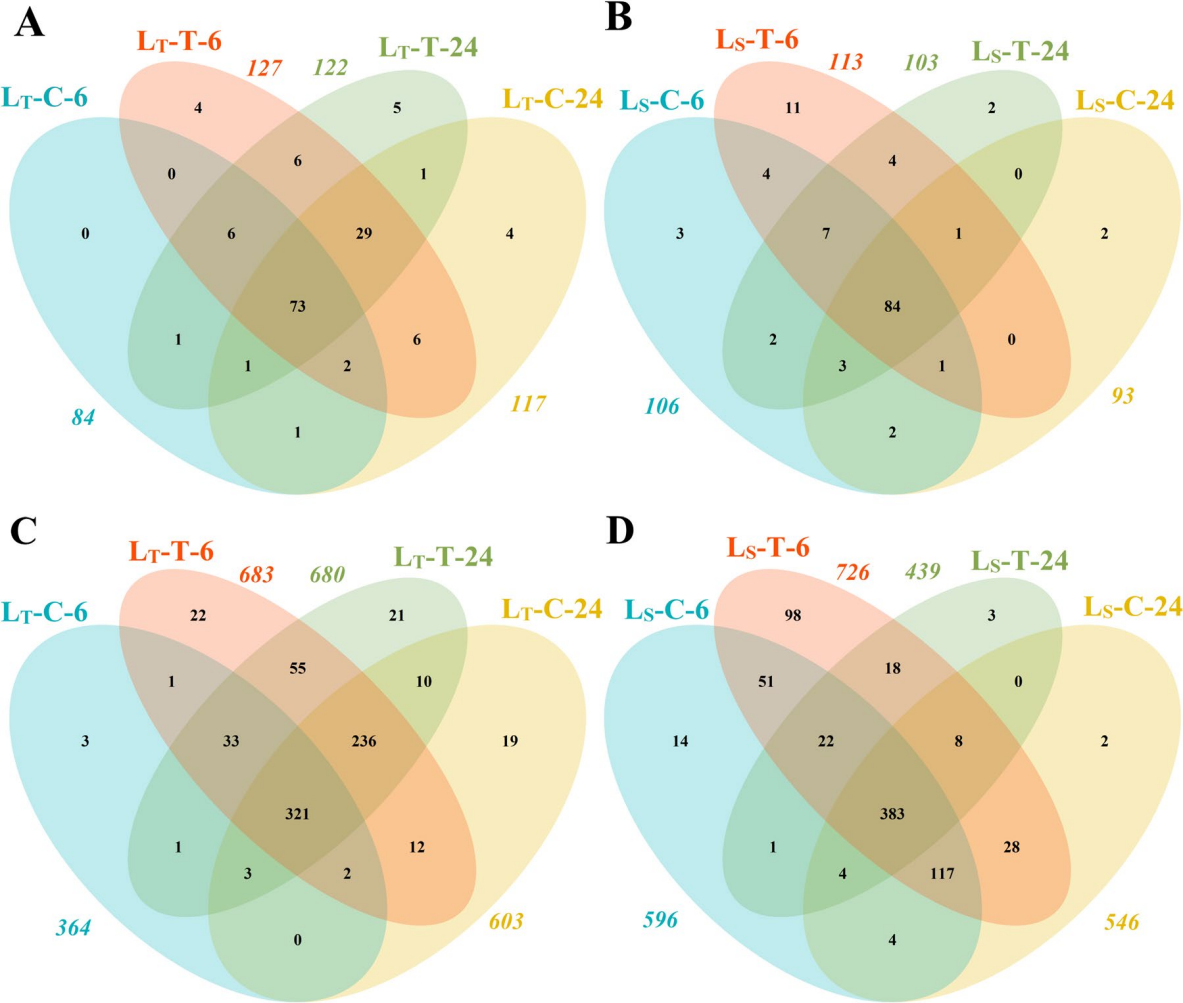
Distribution of miRNAs among genotypes and treatments. (**A**) Known miRNAs in L_T_ under low-temperature treatments and control; (**B**) Known miRNAs in L_S_ under low-temperature treatments and control; (**C**) Novel miRNAs in L_T_ under low-temperature treatments and control; (**D**) Novel miRNAs in L_S_ under low-temperature treatments and control. The total number of miRNAs in each library is shown in italic outside of the diagram

Considering the miRNAs, a total of 145 annotated known miRNAs, belonging to 30 families, were identified (Fig. 4**,** Additional File 3). miRNA families represented by most members were miR166 and miR171 with 12 members, closely followed by miR156, miR164, and miR169 with 11 members. Only 68 miRNAs were present in all eight libraries, most of them belonging to miR156, miR166 (seven members), and miR159 (six members). Even fewer miRNAs were specific to the low-temperature treatment, and none of them were common for both genotypes. For example, zma-miR164e-5p, zma-miR167j-3p, zma-miR169c-5p, zma-miR171c-3p, and zma-miR393b-3p were expressed only during the treatment in L_T_, but not L_s_. Certain miRNAs were expressed only under the optimal conditions, and they were also specific to the genotype.

Additionally, detected known miRNAs were compared to those of different organisms. No matches were found with viruses, bacteria, and animals, but members of 25 miRNA families were expressed in the sequenced libraries of other plant species. miRNA families with the most matching miRNAs were miR171, miR169, and miR395, with over ten matching miRNAs across 35 different plant species. Most common miRNAs were found in *Oryza sativa* (103 matches), *Glycine max* (97 matches), *Populus trichocarpa* (92 matches), *Malus domestica* (91 matches), and *Sorghum bicolor* (90 matches). Additionally, matching miRNAs found across most species were MIR156a (40 plant species), MIR166a (37 plant species), MIR156b (36 plant species). Interestingly, matches were also found among other vascular plants, such as the lycophyte *Selaginella moellendorffii* (12 miRNAs across 6 miRNA families) and bryophyte *Physcomitrella patens* (43 miRNAs across 8 miRNA families).

Unknown sRNAs that could be mapped to the reference sequence were identified as potentially novel miRNAs, and the ones with high confidence included 876 sequences (Fig. 4, Additional File 4). Of the 876 novel miRNAs, 124 were specific to the tolerant genotype and 137 were found only in the sensitive one. In L_T_, 324 novel miRNA sequences were expressed in all four libraries, under the control and treatment conditions, while in L_S_ the same can be said for 386 miRNA sequences. Only 150 novel miRNA sequences were expressed in all eight sequenced libraries. Out of the 189 novel sequences detected only in the treatment libraries (L_T_-T-6, L_T_-T-24, L_S_-T-6, and L_S_-T-24), 27 were common for both genotypes, while 71 were found only in L_T_ and 91 only in L_S_. Analysis of first position nucleotide bias in these miRNA sequences showed that the first nucleotide tended to be adenine (A), while other position biases were not as pronounced. A significant part of these novel miRNAs showed diversified expression levels, and while some showed great abundance (over 1000 reads in at least one of the libraries), most were represented with less than 100 reads. This expression level is lower than that of conserved miRNAs.

### Target prediction of the known and novel miRNAs

A total of 1822 genes were identified as targets for both known and novel miRNAs. Out of 145 known miRNAs, 138 were shown to be able to target 678 genes (Additional File 5), while among the novel miRNAs 1241 potential targets were identified for 440 novel miRNAs (Additional File 6). Nearly 70 target genes were regulated by multiple known and novel miRNAs according to prediction.

GO enrichment analysis showed that most target genes were included in the molecular function of binding (over 57%), particularly protein binding (≈18%), as well as regulation of other biological processes, such as localization and transport (Additional File 7). KEGG analysis on the other hand revealed that most of them were active in different metabolic pathways (≈30%) and biosynthesis of secondary metabolites (≈17%) (Additional File 8). Further gene annotation analysis showed that a significant number of these genes were involved in the plants’ stress response. Many target genes encoded different transcription factors important for the stress response, including heat stress transcription factors (HSF), growth regulating factors (GRF), ethylene responsive factors (ERF), as well as many others such as WRKY, HOX, MYB, bZIP, and auxin-responsive (ARF) transcription factors. Additionally, other genes included in the stress response and signal transduction were identified as targets for these miRNAs, such as genes encoding cysteine-rich protein kinases (RLKs), serine/threonine protein kinases (SAPKs), calcium dependent ion transporters and universal stress proteins (USPs).

### miRNA expression patterns under chilling stress in maize 5-d old seedlings

Differential expression analysis was performed on 933 miRNAs (119 known and 814 novel miRNAs) that had expression levels higher than 5 TPM. Out of the 933 miRNAs, 649 were expressed differentially (DE) between the control and treatment conditions – 89 known and 560 novel miRNAs (Additional File 9).

**Fig. 5:**
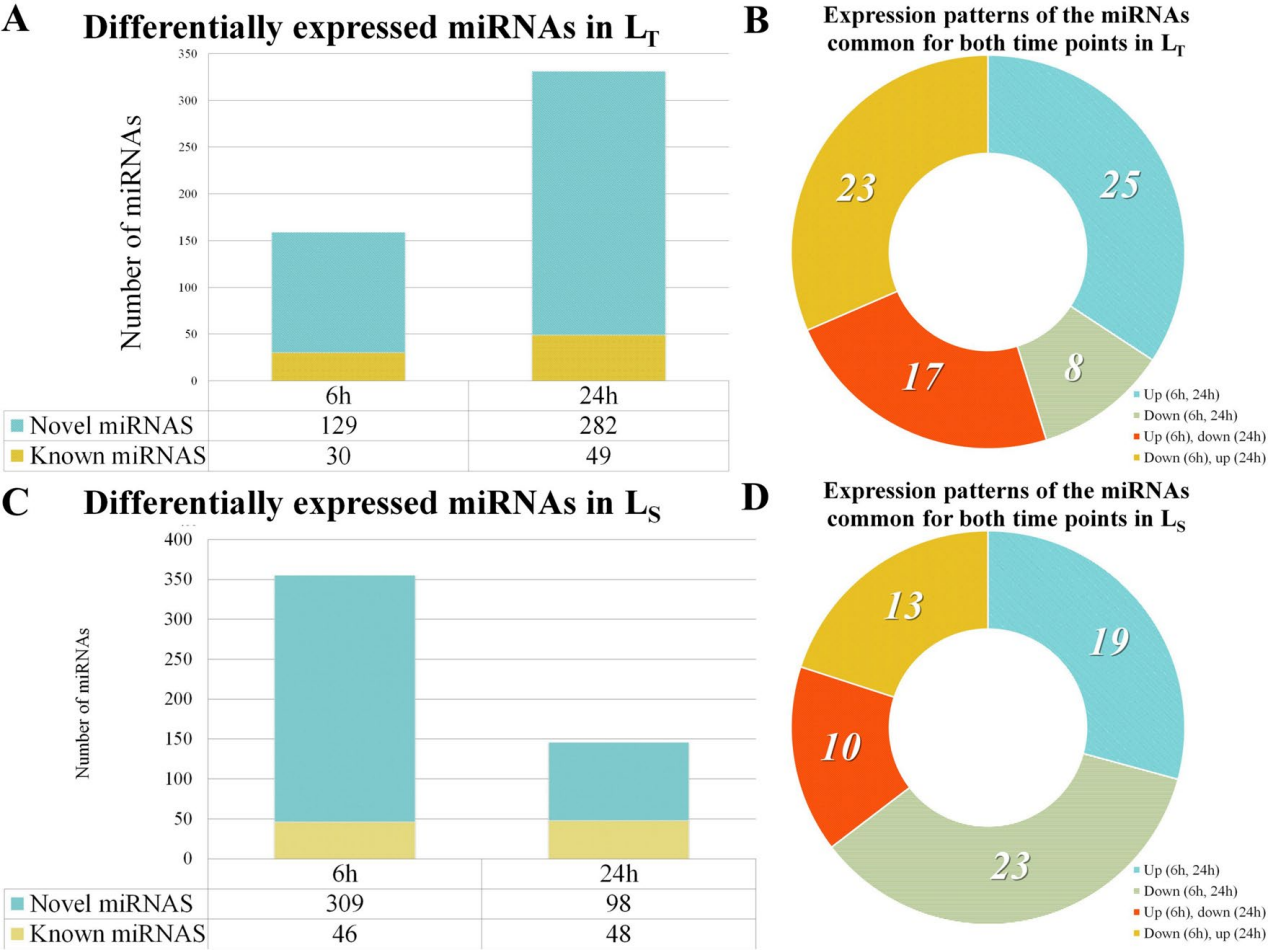
Distribution of differentially expressed miRNAs across the treatments and their expression patterns. (**A**) Differentially expressed known and novel miRNAs after 6 h and 24 h of treatment in L_T_. (**B**) Expression patterns of the 73 miRNAs that are common for both treatment durations in L_T_. (**C**) Differentially expressed known and novel miRNAs after 6 h and 24 h of treatment in L_S_. (**D**) Expression patterns of the 65 miRNAs that are common for both treatment durations in L_S_

In L_T_, there were 417 miRNAs differentially expressed between the control and treatment conditions: 159 after 6 h treatment and 331 after 24 h (Fig. 5A). There were 73 miRNAs common for both treatment durations with various expression patterns (Fig. 5B). On the other hand, among the 436 DE miRNAs in L_S_, 355 were differentially expressed after 6 h treatment and only 146 after 24 h (Fig. 5C). Additionally, the ratio of DE miRNAs with the same expression patterns after both treatment durations was higher in L_S_ compared to LT, 42 in L_S_ (Fig. 5D) and 33 in L_T_ (Fig. 5B).

**Fig. 6:**
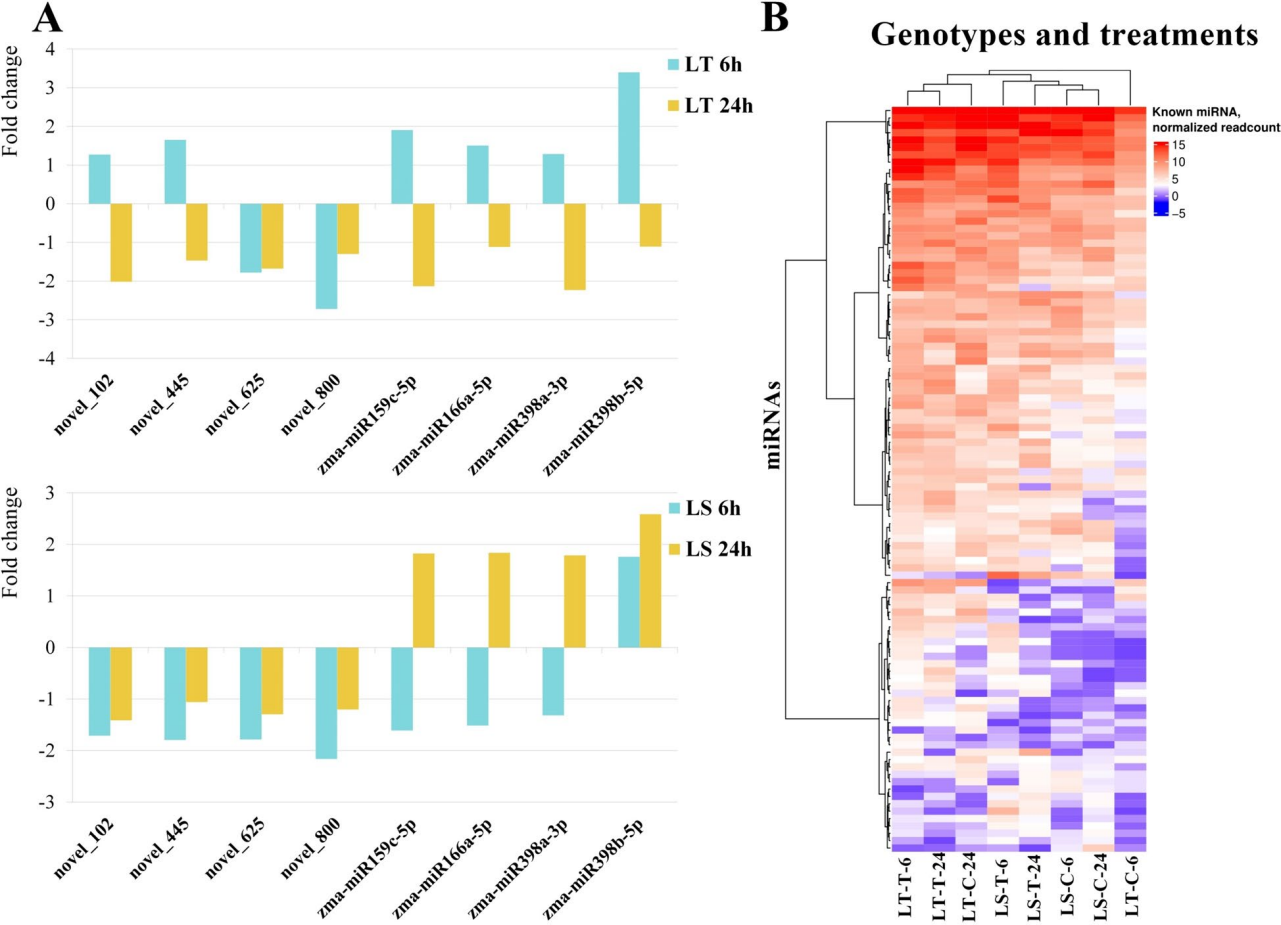
Differential expression of common miRNAs and cluster analysis. **(A)** Differential expression of the eight common miRNAs (novel_102, novel_445, novel_625, novel_800, zma-miR159c-5p, zma-miR166a-5p, zma-miR398a-3p, zma-miR398b-5p), between the control and treated samples in L_T_ and L_S_ after 6 h and 24 h shown as the fold change. (**B**) Cluster analysis, based on the expression patterns of 101 known miRNAs in the eight libraries

A total of 55 DE miRNAs were detected in both genotypes after 6 h of treatment. Twenty-eight followed the same expression pattern – 18 up-regulated and 10 down-regulated. On the other hand, 12 were up-regulated in L_T_ and down-regulated in L_S,_ while 15 were up-regulated in L_S_ but not in L_T_. Considering 24 h treatment, 51 common DE miRNAs were detected. Here, most miRNAs showed the same expression patterns in both L_T_ and L_S_—12 up-regulated and 19 down-regulated. Only eight miRNAs were expressed in both genotypes and at both time points showing different expression profiles (Fig. 6A).

Cluster analysis, based on the expression patterns of 101 known miRNAs, revealed that those expression patterns were genotype-specific, more than treatment-specific. Three clusters were observed – all the samples belonging to L_S_ were classified into a single cluster, while L_T_-C-6 was separated from the rest of L_T_ into a separate cluster. Additionally, the known miRNAs were divided into four clusters based on their expression patterns across all eight libraries. (Fig. 6B).

### Validation of known miRNAs, novel miRNAs and target genes using qRT-PCR

**Fig. 7:**
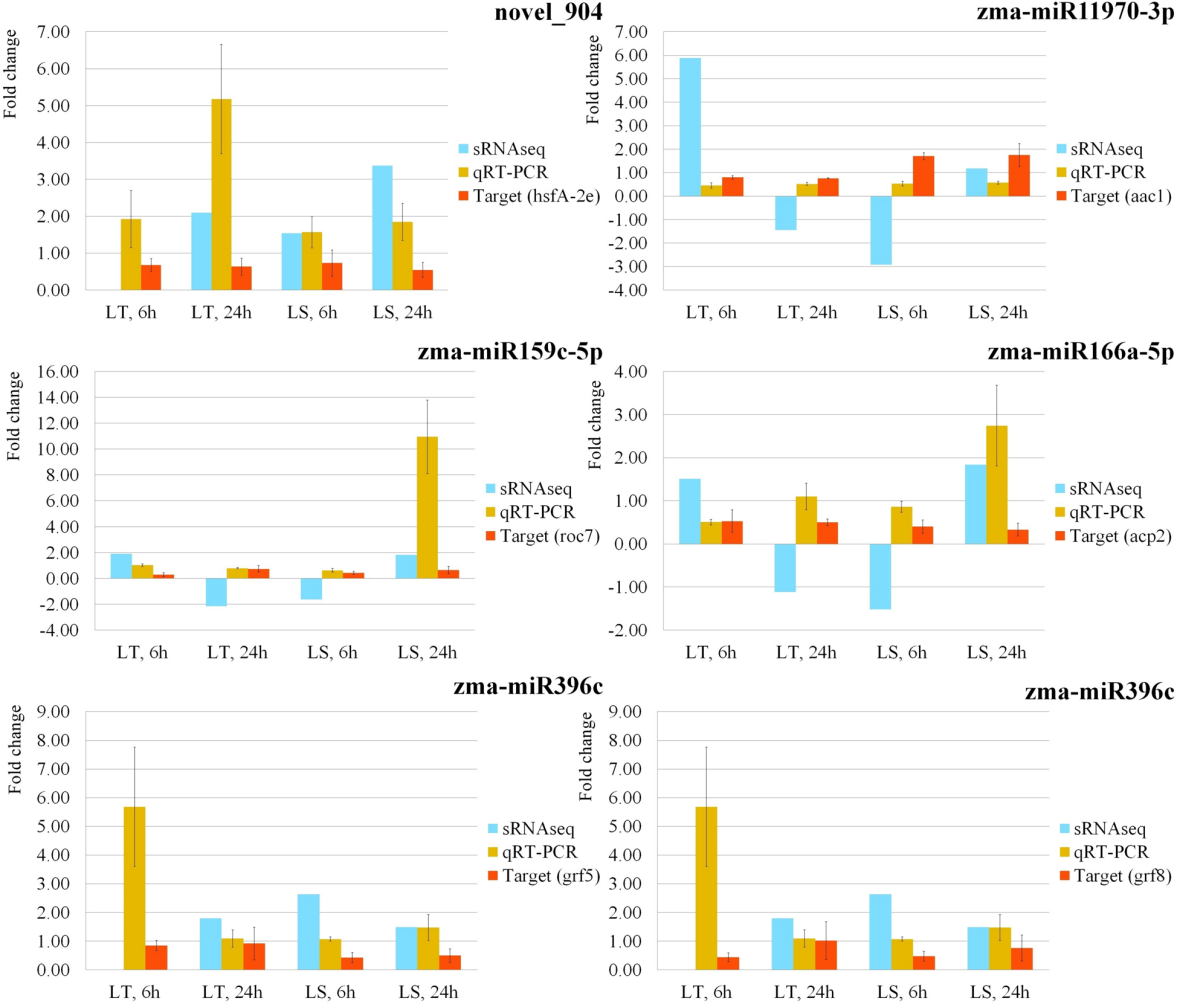
Validation of known and novel miRNAs, as well as their target genes using qRT-PCR. qRT-PCR validation was performed on selected miRNA (novel_904, zma-miR11970-3p, zma-miR159c-5p, zma-miR166a-5p and zma-miR396c), and their targets genes (*hsfA-2e, aac1, roc7, acp2, grf5, grf8*). The expression patterns obtained from the next-generation sequencing (sRNAseq) are shown as the log2 fold changes between the control and treated samples in L_T_ and L_S_ after 6 h and 24 h. qRT-PCR expression patterns are shown as 2.^–ΔΔCt^ values [30, 31] obtained from the ΔΔCt values from control and treated samples in L_T_ and L_S_ after 6 h and 24 h. t-test was performed for mean comparison (*p* < 0.05)

Sequencing reliability was confirmed through qRT-PCR analysis of the miRNA expression levels. Five miRNAs (novel_904, zma-miR11970-3p, zma-miR159c-5p, zma-miR166a-5p and zma-miR396c) were selected for the analysis based on their quantity across all eight libraries and differential expression levels. In general, the expression patterns obtained through RT-PCR matched those obtained through NGS sequencing, with small inconsistencies. Additionally, the expression of certain target genes (*hsfA-2e, aac1, roc7, acp2, grf5, grf8*) was evaluated in the same way and opposite expression profiles between the target genes and their corresponding miRNAs was observed. The results of the validation are shown in Fig. 7.

Additionally, the expression levels of several known *ZmCOI* genes were analyzed using qRT-PCR, at the same time points (Additional file 10). These included: fatty acid desaturases 2 and 7 (*FAD2, FAD7*), dehydration-responsive element binding protein 2A (*ZmDREB2A*), calcium-dependent protein kinase 1 (*ZmCPK1*) and zinc finger protein AN13 (*ZmAN13*). The expression of the *ZmCOI* genes was analyzed at the same time points. Statistically significant differences were found between the control and treatment, as well as between the genotypes, at either of the time points for all five genes.

## Discussion

Chilling stress tolerance in maize seedlings is important for implementing early sowing as a strategy for avoiding the negative consequences of climate change on maize yield and biomass. In recent years, many studies have shown that miRNAs play important roles in the response to abiotic stress in plants. Still, miRNA studies in maize seedling tolerance to chilling stress are scarce, and understanding their mode of action would be a valuable reference for future molecular studies and improvement of breeding strategies. This is the first study designed to investigate the role of 5-d old maize seedlings׳ chilling-responsive miRNAs, their expression patterns, associated target genes and possible functions.

### miRNA in maize compared to other species

Identified 5d-old maize miRNAs (zma-miRNA) were compared to those of other species. Size distribution analysis showed that most abundant miRNAs were 24 nt long. This is consistent with the results from other research focused on zma-miRNA identification under abiotic [33] and biotic [34] stress conditions, as well as during germination [35].

By comparing the identified miRNAs miRNAs from other species, it was confirmed that the most conserved ones belonged to miR156, mir166, miR169, miR395 and mir396 families as they were found in a broad range of plant species, from bryophytes to eudicots [36]. This concurrence shows that many miRNAs belong to evolutionary conserved regulatory modules that play important roles in plant development [37]. Expectedly, maize shares most of the common miRNAs with other monocots such as rice and sorghum. But, common miRNAs were also found with soybean and tree species (such as *Malus domestica* and *Populus trichocarpa).* This suggests a possibility that many miRNAs had independent origins, unrelated to their phylogenetic tree during evolution, meaning that they could be homoplasious rather than homologous.

### Roles of zma-mirRNA in the response to chilling stress

Out of the 933 analyzed miRNAs, 98 were differentially expressed in both genotypes at one of the time points – 55 after six hours and 51 after one day of exposure to the treatment conditions. Only eight were differentially expressed in both genotypes at both time points (novel_102, novel_445, novel_625, novel_800, zma-miR159c-5p, zma-miR166a-5p, zma-miR398a-3p, zma-miR398b-5p). Over 300 genes were found to be potential targets of these 98 miRNAs.

GO enrichment analysis revealed that target genes were involved in various biological processes, mainly in different aspects of growth and development (zma-miR396, zma-miR156, zma-miR319, and zma-miR159), and in the direct response to stress factors. Of these, some were involved in the antioxidative response (zma-miR398), signal transduction (zma-miR156, zma-miR167, zma-miR169) and regulation of water content (zma-miR164, zma-miR394, zma-miR396). The rest of the targeted genes had roles in cell wall formation, photosynthesis, protein metabolism, and nutrient assimilation.

#### zma-miRNAs might regulate growth and organ development during chilling stress

Various miRNAs target genes that encode different components of regulatory networks involved in plant growth, root and shoot development, tissue morphogenesis, and other developmental processes. miRNA families known to be involved in this process are miR396, miR156, mir166, miR319, and miR159.

The role of miR396 is in controlling the growth of multiple tissues and organs in a variety of species by modulating growth regulating (GRFs) and GRF-interacting factors (GIFs) [38]. The miR396–GRF/GIF module fulfills multiple roles in plant development. They include the promotion of shoot and shoot lateral organ growth, particularly leaf development and leaf cell proliferation, as well as overall cell proliferation and expansion. Considering miR156 family members, they are key regulators of squamosa promoter-binding (SPB) like proteins (SPL) expression, controlling the timing of vegetative phase change, leaf initiation rate, shoot branching, and lateral root development [39, 40]. The role of miR319 is important for maintaining cell division functions in the leaf meristem, through expression regulation of several transcription factors (TF) such as gibberellin-and-abscisic-acid-regulated MYB (GAMYB), and teosinte branched1/cycloidea/PCF (TCP) [41]. Besides miR319, miR159 also participates in GAMYB regulation by suppressing its expression during development, ensuring normal growth during the vegetative stage [42]. It was also shown that miR159 was accumulated in response to different abiotic stresses in various crop species and that this increase seems to be regulated by abscisic acid (ABA). Our results of the target prediction analysis coincide with the miRNA-target gene connections found in the literature, giving further support to these regulatory networks. Furthermore, target prediction showed a potential role for zma-miR528a and zma-miR408a in root development regulation, with miR528a possibly controlling bHLH TFs necessary for root hair development and miR408a being involved in Casparian strip lignification and root cell differentiation.

Differential expression analysis showed that the expression of the identified miRNAs was affected by low temperatures, with many being up-regulated in both genotypes after 6 h (zma-miR156d-5p, zma-miR156k-5p) or 24 h (zma-miR319a-3p, zma-miR396c). Other plant species also showed matching expression profiles under abiotic stresses. It was shown that miR396 are up-regulated by various stress conditions, such as drought and UV-B irradiation in *Arabidopsis* [43]. Similarly, miR156 expression was increased in many plant species during different abiotic stress treatments, including heat [44], high salinity [45], and drought [46].

The described expression profiles indicate the potential role of the analyzed miRNAs in inhibiting seedling growth and development under chilling conditions, suggesting the up-regulation of the specific miRNAs and the consequential decrease in the expression of their target genes is an important aspect of the plant’s survival strategy.

#### zma-miRNAs involved in antioxidative response regulation

The miR398 are known regulators of the antioxidative response in plants, controlling the expression of Cu/Zn superoxide dismutase 1 and 2 (*CSD1* and *CSD2*) which are responsible for reactive oxygen species (ROS) scavenging [47]. Expression of miR398 is regulated by ABA, as well as by WRKY TFs which are known to be involved in abiotic stress responses, particularly in chilling [48]. Two miR398 family members (zma-miR398a and zma-miR398b) showed a significant difference in their expression levels, at both time points and in both genotypes. In L_S_, the expression of zma-miR398b was increased at both time points and of zma-miR398a after 24 h. Contrary to L_S_, the initial up-regulation of both miRNAs after 6 h was followed by down-regulation after 24 h in L_T_. Down-regulation of miR398 is related to abiotic stress tolerance and is followed by the increase in *CSD1* and *CSD2* expression in different plant species under many stress treatments [49, 50]. This is concurrent with the DE analysis performed for L_T_ and L_S_. To our knowledge, this is the first report of miR398 being differentially expressed during chilling stress in maize.

Additionally, according to target prediction, several potentially novel miRNAs could also be involved in antioxidative response. For example, novel_16, which is possibly involved in regulating 2-alkenal reductase necessary for alleviating oxidative stress in maize [51], was up-regulated in both genotypes after 24 h. On the other hand, novel_447 which could be involved in regulating the synthesis of ascorbic acid by targeting L-gulonolactone oxidase 2 and in hydrogen peroxide removal by targeting L-ascorbate peroxidase 2, was down-regulated after 6 h in both genotypes. Both enzymes are involved in the abiotic stress response and alleviating the negative effects of ROS [52], indicating that the down-regulation of this miRNA could be related to increased resistance to oxidative damage during chilling.

#### zma-miRNAs included in stress signal transduction

Target prediction analysis showed that several DE zma-miRNAs (zma-miR156d-5p, novel_447, zma-miR528a-5p, and novel_342) have a role in Ca^2+^ signal transduction, known to play a critical role in chilling stress response in plants. zma-miR156d-5p and novel_447 are involved in the regulation of calmodulin-binding transcription activator 3 (CAMTA3), which perceives an increase in calcium level as a response to chilling and freezing stress [53]. CAMTA3, together with CAMTA1, CAMTA2, CAMTA4 and CAMTA5, is required for the chilling-induced expression of TFs of chilling regulated (*COR*) genes *DREB1B/CBF1*, *DREB1C/CBF2* and *GOLS3* [54]. Büyük et al. predicted that among others, miR156 potentially regulates CAMTA factors in various plant species [55]. Several studies noted the potential role of miR156 in regulating several CAMTA during periods of abiotic stress, but not in maize under low temperature conditions. Additionally, Kansal et al. showed that calcium cytosolic levels, through CAMTA4 and CAMTA6, play a role in regulating the expression of os-miR156a and os-miR167h under drought conditions in rice and this could indicate a possibility that a similar relation also occurs in maize [56].

Zhao et al. suggested that the role of miR156 during abiotic stress could be in balancing the vegetative development and chilling or freezing response [57]. Additionally, it was shown that miR156 and their SPL-targets contribute to the activation of ABA signaling pathways, pointing also to their involvement in the chilling stress response [58]. Expression of miR156 after exposure to low temperatures varies among species. Accordingly, down-regulation of miR156 is related to an increase in tolerance in rice [59], but overexpression of miRna156 improved chilling tolerance in tobacco [60]. Herein, zma-miR156d-5p was up-regulated in maize 5d old seedlings of both genotypes after 6 h.

Another two identified miRNAs involved in stress signal transduction, zma-miR528a-5p and novel_342, play a role in regulating calcium-dependent protein kinase 27 (CDPK27). It is well known that CDPKs are major regulators of stress responses in plants, directly regulating target proteins. A study in tomato plants showed that CDPK27, among other CDPKs, can induce crosstalk between ROS and other signaling molecules, leading to the activation of ABA. Silencing of CDPK27 significantly decreased the ROS accumulation, as well as mitogen-activated protein kinases (MPK1/2) further downstream. This suggests that CDPK27 may play a crucial role in adaptation to chilling stress [61]. miR528 have been shown to have a role in various abiotic stresses, such as heat in wheat [62], excess nitrate in maize [63], and drought in rice [61]. Aydinoglu showed that miR528 are up-regulated under chilling conditions in the maize leaf meristem in the later developmental stages [64]. On the contrary, our results show that in the 5-day old seedling stage, their down-regulation is linked to cold tolerance—differential expression showed different expression patterns of zma-miR528a-5p and novel_342 after 24 h in the analyzed genotypes – significant down-regulation in L_T_, but up-regulation in L_S_.

#### Regulation of water loss by zma-mirRNA during chilling stress treatment

Chilling stress is known to invoke similar responses in plants as droughtsince the capacity of water absorption and uptake decreases with temperature decline. Both low temperatures and drought cause an imbalance in the water status and in the short term lead to an upsurge of ABA that promotes stomatal closure and in the long term lead to a decrease in leaf area and stomatal density [65].

Lower transpiration and higher water uptake efficiency (WUE) were associated with reduced stomatal density in the leaf abaxial epidermis, as well as with higher expression of negative stomatal development regulator STOMATAL DENSITY AND DISTRIBUTION 1 (*SDD1*) in *Arabidopsis* [66]. Target prediction revealed that several members of zma-miR164 family, as well as zma-miR394a-5p, are involved in regulating *SDD1* expression in maize 5-d old seedlings. While zma-miR394a-5p was up-regulated in both genotypes, zma-miR164f-3p was significantly down-regulated after 24 h in L_T_. The role of miR164 in drought stress and water uptake regulation is well known, but mostly regarding their involvement in stress response through NAC TF modulation [67]. However, their possible role in regulating *SDD1* has not been previously described. Herein, it was shown that zma-miR164f could have a role in developing chilling and other abiotic stress tolerance in maize.

The nuclear factor Y (NF-Y) complex is induced by abiotic stressors [68]. Yang et al. showed that NF-Y affects drought tolerance through modulation of ABA signaling pathway and further downstream the modulation of stomata movement, osmolyte accumulation, and ROS metabolism [69]. miR169 respond to and regulate NF-Y in many plant species under different abiotic stress factors [68]. This has also been shown in maize [70], but not under chilling stress. Herein, zma-miR169i-5p was down-regulated in both genotypes after 6 h. Evidence on the role of miR169 in establishing abiotic stress tolerance is conflicting. In some cases, the overexpression of miR169 resulted in increased tolerance [71], but in others, it was vice versa [68]. This suggests that the roles of miR169 in regulating abiotic stress response are more complex and require further research.

Additionally, according to target prediction, zma-miR396c was involved in regulating the protein import into the nucleus in response to chilling by modulating the expression of KPNB1, a homolog of human importin β. KPNB1 acts as a negative effector of drought tolerance in *Arabidopsis* and its inactivation leads to increased stomatal closure in response to ABA and water loss reduction [72]. While KPNB1 was up-regulated in both genotypes after 24 h, its expression level was higher in L_T_. The possible role of miR396c in regulating nucleus import during low temperature stress has not been previously described, but their role in drought and other abiotic stress responses is known [73]. As with miR164, there are conflicting results when it comes to the effect of miR396c on abiotic stress tolerance – in some cases, its overexpression led to the increase in tolerance [74], but in the others, tolerance was acquired by down-regulating miR396 [75]. This suggests that the expression profiles and regulatory machinery behind them could vary on the plant species. Based on their role in regulating leaf development and leaf cell proliferation [76], the role of miR396 in regulating water loss could be in decreasing leaf surface and decreasing transpiration.

**Fig. 8:**
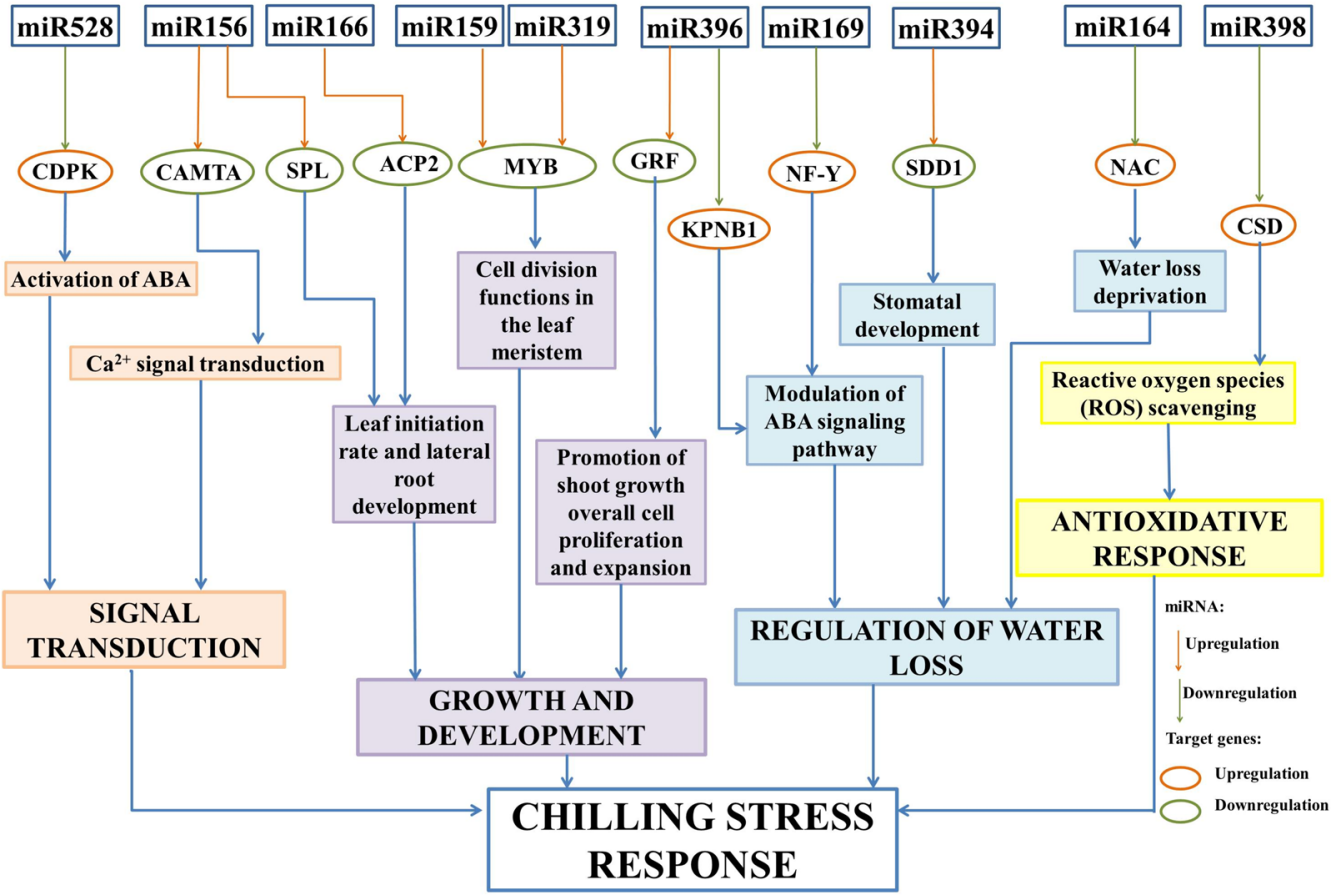
Potential regulatory network of the DE miRNAs and their target genes involved in the maize 5d-old seedlings’ chilling response. Potential regulatory network includes the miRNA (miR156, miR159, miR164, miR166, miR169, miR319, miR394, miR396, miR398, and miR528) and their target genes *(CDPK, CAMTA, SPL, ACP2, MYB, GRF, KPNB1, NF1, SDD1, NAC*, and *CSD*) involved in signal transduction, growth and development, waterloss regulation and antioxidative response. Downregulation of a miRNA and/or target gene is labeled green, while the upregulation is labeled orange

Based on the information discussed above, the potential regulatory network of the miRNAs (miR156, miR159, miR164, miR166, miR169, miR319, miR394, miR396, miR398, and miR528) and their target genes involved in the maize 5d-old seedlings response to chilling is presented in Fig. 8.

## Conclusion

Early sowing of maize is projected to reduce the negative effects of climate change and the development of genotypes tolerant to low-temperature stress in early stages of plant growth and development can be considered to be of vital importance. miRNAs are regarded as a promising strategy for complex trait improvement, including yield, biomass production, and stress tolerance. This study presents a first report on the study of miRNAs and their targets involved in chilling stress tolerance of 5d-old maize seedlings. In order to identify miRNAs and understand their potential functions, eight small RNA libraries of a tolerant and sensitive inbred line were constructed and sequenced. Thirty-three known and 65 novel zma-miRNAs, differentially expressed in response to chilling stress, were identified. Target prediction, GO, and KEGG-based enrichment analysis showed that many of the identified miRNAs affect genes involved in regulating plant growth and organ development, stress signal transduction, antioxidative response, and water loss regulation under stress. This research provides a significant basis for further network analysis, with other coding and non-coding RNAs, as well as functional analyses. Furthermore, the findings provide important information for the identification of chilling-responsive genes which could be beneficial for molecular breeding of chilling-tolerant maize.

### Supplementary Information

Supplementary Material 1.

Supplementary Material 2.

Supplementary Material 3.

Supplementary Material 4.

Supplementary Material 5.

Supplementary Material 6.

Supplementary Material 7.

Supplementary Material 8.

Supplementary Material 9.

Supplementary Material 10.

## Data Availability

Sequence data generated during the current study are available in the The European Nucleotide Archive (ENA) (https://www.ebi.ac.uk/ena/browser/home). The data are submitted under the project name “Maize sRNAs involved in cold stress response in 5-day old seedlings” (Accession number: PRJEB68228), with the sequences of each individual library submitted under accession numbers ERR12245651—ERR12245658.
