# Supplementary figures and images for: Maize miRNAs and their putative target genes involved in chilling stress response in 5-day old seedlings
Source: BMC Genomics. 2024 May 15;25:479. doi: 10.1186/s12864-024-10403-1 (PMC11094857; doi:10.1186/s12864-024-10403-1)

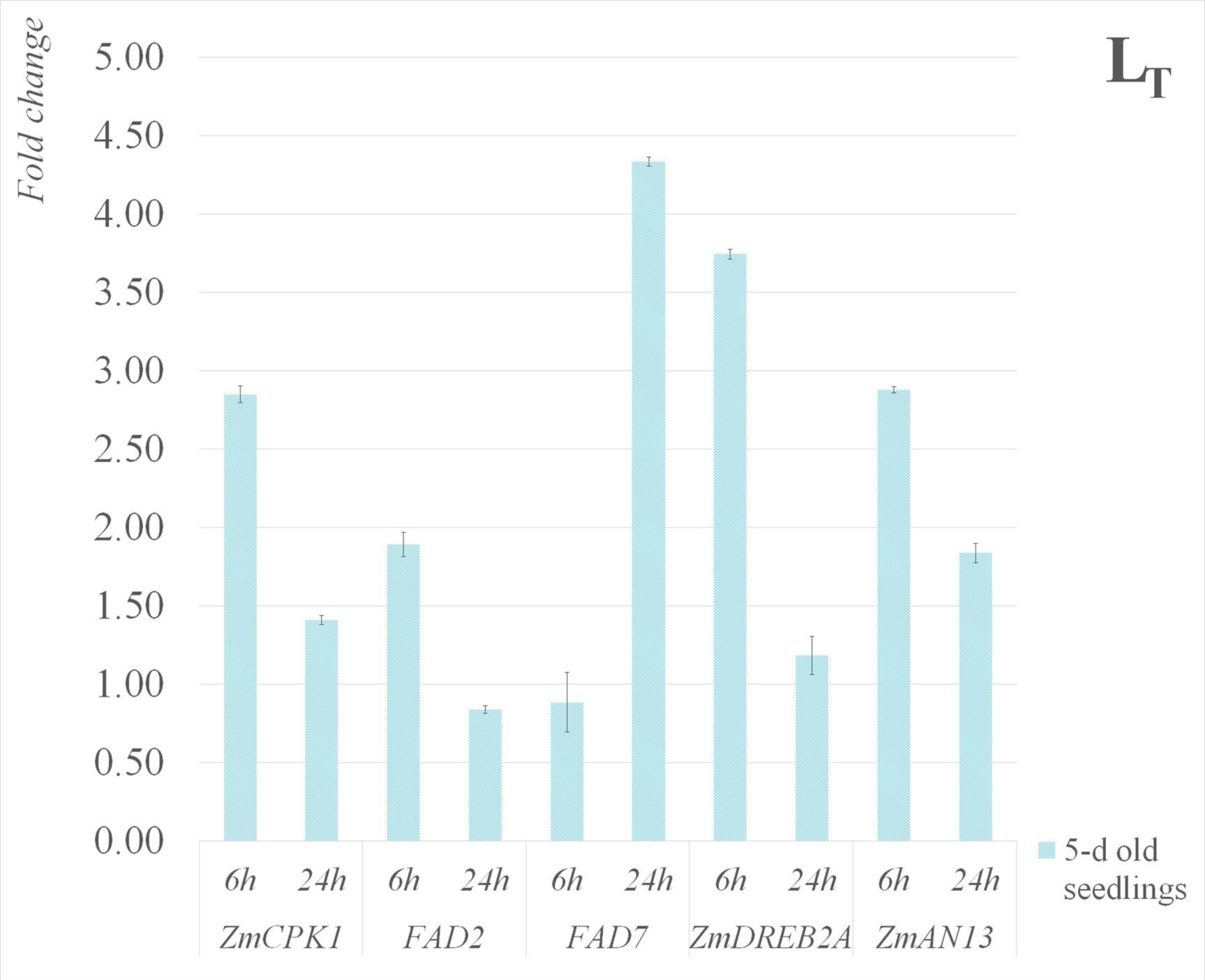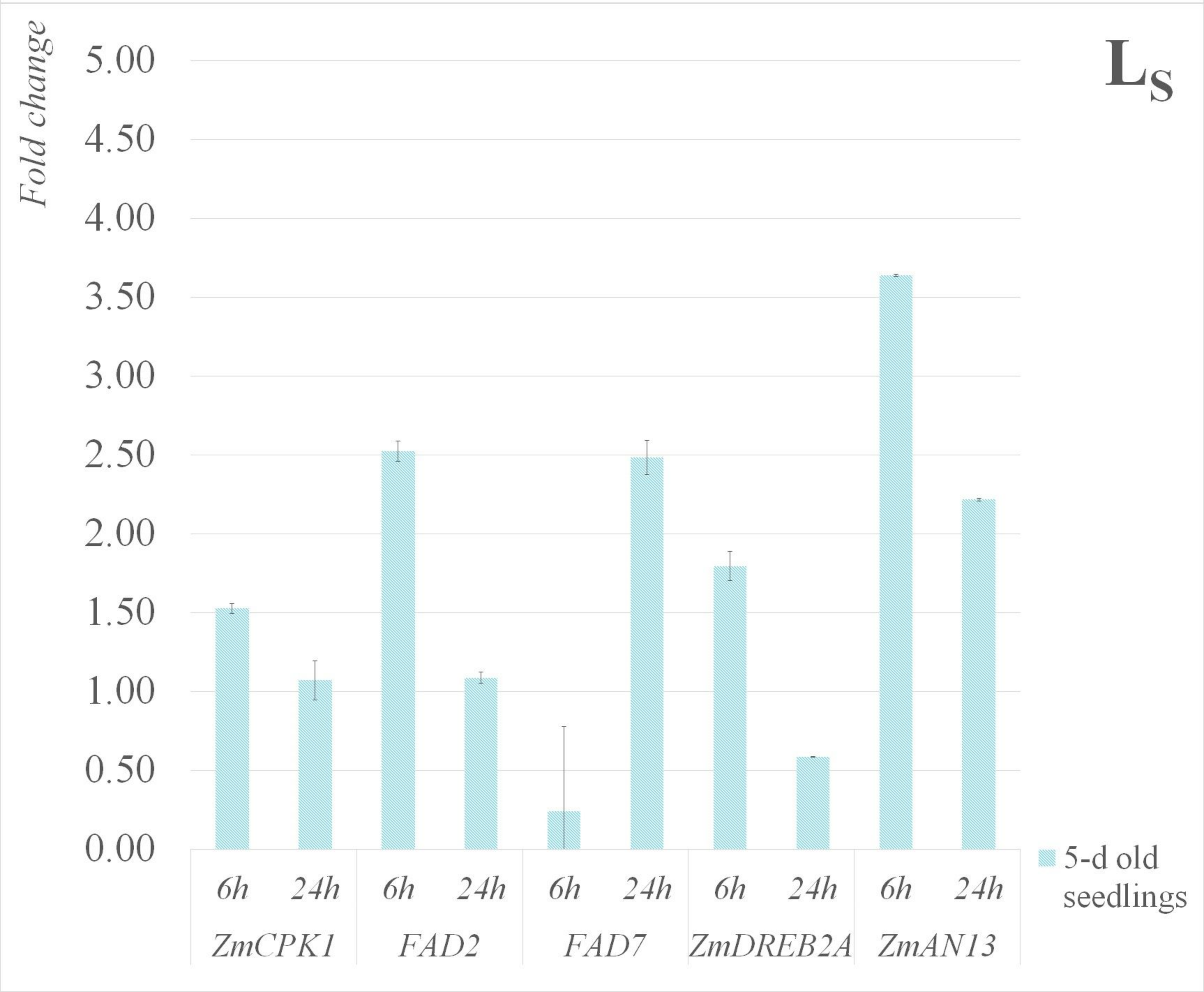

Supplement: Supplementary file 10 — Supplementary Material 10. [file 12864_2024_10403_MOESM10_ESM.pdf]
